# A Pyrido‐Quinoxaline Derivative That Downregulates Reticulon 3 Protein Exhibits Potent Antiviral Activity Against Zika Virus

**DOI:** 10.1002/jmv.70605

**Published:** 2025-09-18

**Authors:** Erika Plicanti, Andrea Deiana, Silvia Nottoli, Giulia Lottini, Roberta Ibba, Sandra Piras, Carlo Di Marzo, Silvia Vegni, Michele Lai, Mauro Pistello, Antonio Carta, Giulia Freer

**Affiliations:** ^1^ Department of Translational Research and New Technologies in Medicine and Surgery, Retrovirus Center University of Pisa Pisa Italy; ^2^ Department of Medical Biotechnologies University of Siena Siena Italy; ^3^ Department of Medicine, Surgery and Pharmacy University of Sassari Sassari Italy; ^4^ Pisa University Hospital Azienda Ospedaliera Universitaria Pisana Pisa Italy

**Keywords:** antiviral activity, endoplasmic reticulum, Flaviviridae, pyrido‐quinoxaline derivatives, reticulon 3, Zika virus

## Abstract

In the wake of the COVID‐19 pandemic, awareness of emerging pathogens has significantly increased, prompting greater investment in research and preparedness. In this context, arboviral diseases are recognized as unmet medical challenges due to their rapid spread. Notably, the geographical range of several flaviviral diseases is expanding: Zika virus (ZIKV), a member of the *Flaviviridae* family, has recently been linked to outbreaks associated with a rise in microcephaly cases in tropical regions. To contribute to the development of novel antiviral therapies, evaluation of a set of compounds with an antiviral activity against ZIKV was carried out. These compounds were originally identified as inhibitors of bovine viral diarrhea virus, another member of the *Flaviviridae* family. Two related compounds turned out to be active against ZIKV. One emerged as a particularly strong antiviral candidate, demonstrating high efficacy in inhibiting ZIKV replication, and became the focus of this study. Its activity was tested against a number of viruses of human health relevance and the compound was found to be effective against a number of viruses that use the endoplasmic reticulum as a replication hub. Indeed, we found that the Reticulon 3 protein is potently downregulated in the presence of the compound, whereas other endoplasmic reticulum‐resident proteins are not affected. Because Reticulon 3 has a role in the replication of positive‐sense single‐stranded RNA viruses, an indirect antiviral effect of the compound studied was hypothesized. This compound may be considered as a promising lead for further studies aimed at the development of broad‐spectrum antiviral drugs.

**Abbreviations**
BVDV: bovine viral diarrhea virus
CC_50_
: half maximal cytotoxic concentration
CHIKV: chikungunya virus
CVBx: Coxsackie B virus, serotype x
DENV: dengue virus
EC_50_
: half maximal effective concentration
EIDD‐1931: β‐d‐N4‐hydroxycytidine
ER: endoplasmic reticulum
HCV: hepatitis C virus
HSV2: herpes simplex virus 2
IAV: influenza A virus
MOI: multiplicity of infection
OD: optical density
ON: overnight
PFU: plaque‐forming unit
p.i.: post infection
qRT‐PCR: quantitative reverse transcriptasepolymerase chain reaction
RBV: Ribavirin
RO: replication organelle
RTN: reticulon
SI: Selectivity Index
SOF: Sofosbuvir
ssRNA+: positive‐sense single stranded RNA
TCID_50_
: 50% tissue culture infectious dose
TOA: time‐of‐addition
TOSV: Toscana virus
USUV: Usutu virus
VSV: vesicular stomatitis virus
VV: vaccinia virus
VV‐IND‐G: vaccinia virus recombinant for VSV‐Indiana G protein
WNV: West Nile virus
ZIKV: Zika virus

## 1 Introduction

The world has faced devastating pandemics from emerging or mutating viruses, with arboviruses posing significant challenges due to their rapid evolution and host range expansion. A notable example is Zika virus (family *Flaviviridae*, genus *Orthoflavivirus*, ZIKV), discovered in 1947 as a mild disease spread by *Aedes aegypti* mosquitoes [1]. It gained attention during outbreaks in French Polynesia and Brazil, where it evolved from causing mild illness to severe neurological conditions, like Guillain–Barré Syndrome and microcephaly [2, 3, 4]. Similarly, West Nile virus (family *Flaviviridae*, genus *Orthoflavivirus*, WNV) and Usutu virus (family *Flaviviridae*, genus *Orthoflavivirus*, USUV) have become endemic in regions like North America and Southern Europe, with WNV as a leading cause of mosquito‐borne meningoencephalitis in the United States [5]. In Europe, until 2021, 16 countries had reported WNV infection cases, mostly in the South [6].

*Flaviviridae* are positive‐sense single‐stranded RNA (ssRNA+) viruses; as such, they reorganize the endoplasmic reticulum (ER) into replication organelles (ROs) to shield their genome transcription from cellular defences [7]. Cellular Reticulon 3 (RTN3) and viral nonstructural proteins NS4A and NS4B, which bend the ER membrane, are involved in this process. The first is an ER resident protein involved in membrane curvature in tubule tips [8], whereas NS4A and NS4B have several roles, among which membrane remodeling and induction of autophagy [9, 10]. While RTN3 inhibits Hepatitis C virus (HCV) [11], it favors replication of viruses like SARS‐CoV‐2 and enterovirus 71 [12, 13]. Recent studies highlight that RTN3 is also crucial for ZIKV replication and targeting it may offer a broad antiviral strategy [14, 15]. Interestingly, Vaccinia virus (VV), belonging to *Poxviridae*, the only dsDNA virus family that replicates in cell cytoplasm, was also demonstrated to depend on ER remodeling for its replication [16]. Indeed, VV uses ER‐remodeling mechanisms similar to those of ZIKV, relying on RTN‐like proteins to drive membrane curvature essential for virogenesis [17].

**Figure 1:**
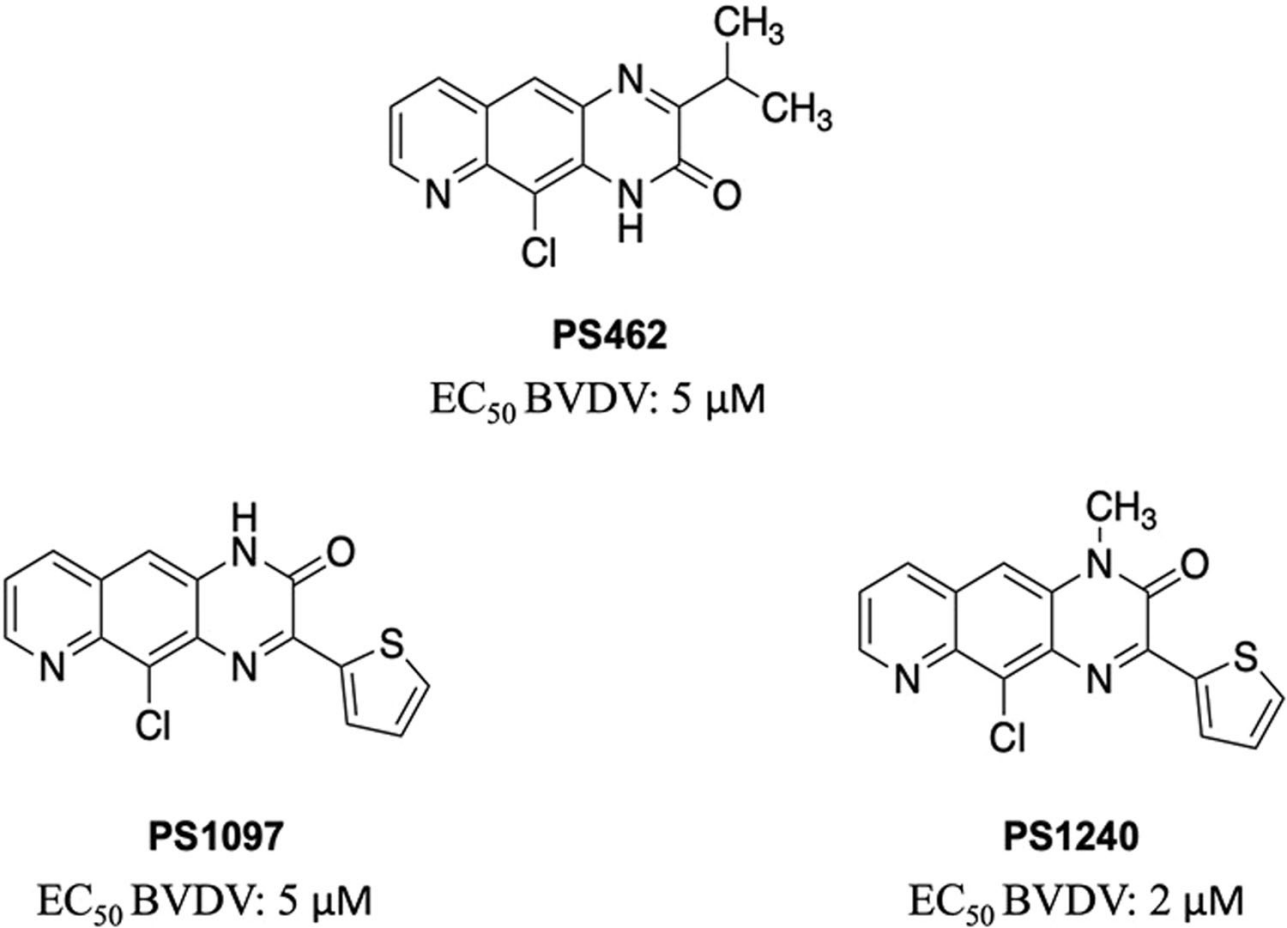
The 4 pyrido[2,3‐g] quinoxalinone derivatives studied here for the anti‐ZIKV activity. Compound **PS462,** previously named 4 h, used as a negative control and active compounds **PS1097** and **PS1240**, previously named 4 and 18, respectively. [18]

The COVID‐19 pandemic has renewed interest in antiviral drug development, focusing on both direct and indirect approaches. Direct‐acting agents target viral enzymes or proteins, while indirect ones disrupt the cellular pathways viruses exploit. The convergence of replication strategies across diverse viruses underscores the potential of targeting these shared pathways in antiviral drug development. In this study, a library of 24 compounds previously shown to inhibit Bovine Viral Diarrhea Virus (family *Flaviviridae*, genus *Pestivirus*, species *Pestivirus bovis*, BVDV) and Coxsackievirus B5 (family *Picornaviridae*, genus *Enterovirus*, species *Enterovirus betacoxsackie*, CVB5) was tested against ZIKV [18, 19, 20, 21, 22] (Table S1). Of these, 15 showed activity, with Effective Concentration 50 (EC_50_) values between 0.69 and 100 µM. Two pyrido‐quinoxaline derivatives (Figure 1) displayed good antiviral activity, with **PS1097** emerging as the most promising. Its low EC_50_ and high Selectivity Index (SI) highlighted its potential as a broad antiviral agent and provided valuable insights into its mechanism of action.

## 2 Materials and Methods

### 2.1 Chemical Compounds

Compound synthetic routes and characterization are reported in Supporting Information: Material S1 and in [18, 19]. Starting materials were purchased from Merck‐Sigma‐Aldrich and Fluorochem. Ribavirin[1‐(b‐d‐ribofuranosyl)‐1H‐1,2,4‐triazole‐3‐carboxamide, RBV), β‐D‐N4‐hydroxycytidine (EIDD‐1931, Merck) and Sofosbuvir (SOF; GS‐7977; MedChemExpress) [23]. were used as reported by manufacturers.

All compounds were stored at 10 mM in DMSO at room temperature for 1 month. For longer storage, aliquots were kept at −20°C.

### 2.2 Cells

A549, Huh‐7 [24], and Vero E6 cells were cultured in Dulbecco's modified Eagle's medium (DMEM) (Gibco), 1 mM glutamine, 1 mM sodium pyruvate, 7% fetal bovine serum (FBS, Gibco). Vero E6 TMPRSS2 (Vero TMPRSS) [25] were cultured like Vero‐E6 cells, with 1 mg/mL of G418 (Merck) added once a month. Mosquito C6/36 cells (ATCC, CCL‐1660) were cultured at 28°C (no CO_2_) in Leibovitz's medium, 1% non‐essential aminoacids, 1 mM glutamine, 1 mM sodium pyruvate, 10% FBS. MDCK cells (ATCC) were grown in Ex‐cell serum‐free medium (Merck), 1 mM glutamine. HMC3 (a gift by Barbara Costa, University of Pisa) were grown in EMEM, 1 mM glutamine, 1 mM sodium pyruvate, 10% FBS. All cells were cultured without antibiotics and checked for *Mycoplasma* as described [26].

### 2.3 Viruses

ZIKV, strain Brazil/2016/INMI1 (ZIKV^Br^) (National Institute for Infectious Diseases Spallanzani, Roma, Italy), ZIKV strain MP1751 (ZIKV^Ug^), WNV strain B956, USUV catalog number 105081 v (Public Health England), Chikungunya virus (family *Togaviridae*, genus *Alphavirus*, species *Alphavirus chikungunya*, CHIKV) strain UVE/CHIKV/2006/RE/LR2006_OPY1(001v‐EVA83) (European Virus Archive) were propagated on Huh‐7 cells. Influenza A virus (family *Orthomyxoviridae*, genus *Alphainfluenzavirus*, species *Alphainfluenzavirus influenzae*, IAV) strain A/PR/8/34 (ATCC) was propagated on MDCK cells. Vesicular stomatitis virus (family *Rhabdoviridae*, genus *Vesiculovirus*, species *Vesiculovirus Indiana*, VSV) (courtesy of Guido Antonelli, University La Sapienza, Rome) and Vaccinia virus recombinant for VSV‐Indiana G protein (family *Poxviridae*, genus *Orthopoxvirus*, species *Orthopoxvirus vaccinia*, VV‐IND‐G), (courtesy of Rolf Zinkernagel, University of Zurich, Switzerland) [27] were propagated on Vero E6 cells. CVB5 (Unit of Virology, AOUP Pisa, Italy) was propagated on HeLa cells. Toscana Virus (family *Bunyaviridae*, genus *Phlebovirus*, species *Phlebovirus toscanaense*, TOSV, clinical isolate courtesy of Grazia Cusi, University of Siena, Italy) was propagated on C6/36. SARS‐CoV‐2 VR PV10734 (GISAID EPI_ISL_2544194) (family *Coronaviridae*, genus *Betacoronavirus*, species *Betacoronavirus pandemicum*, SARS‐CoV2‐Mi), courtesy of Università San Raffaele, Milan, Italy, was propagated on VERO‐TMPRSS cells.

### 2.4 Determination of Viral Yield Reduction and Effective Concentration 50%

The virus yield reduction assay was performed as described by Prichard [28] (Table 1). A primary 96‐well plate with 1 × 10^4^ Huh‐7 cells/well was infected with ZIKV at a multiplicity of infection (MOI) of 5 for 2 h at 37°C, 5% CO_2_. The inoculum was replaced with 100 μL 1:2 dilutions of each compound in DMEM, 2% FBS, starting from 100 μM. Cells were incubated for 48 h then viral yields in supernatants were titrated. For ZIKV, supernatants were diluted 1:3 and added onto Vero E6 monolayers and removed after 2 h at 37°C post infection (p.i.). The inoculum was replaced with 100 μL of 1% Carboxymethyl cellulose (Merck) in complete medium. After 72 h, cells then fixed with 4% buffered formalin solution (Merck) and stained with 1% crystal violet (Merck). Titers were calculated as follows:

PFU/mL = number of plaques × 3^*n*^/vol infection where n: dilution factor at which plaques were counted.

For plaque reduction assays, A549, Vero E6 or Huh‐7, 3 × 10^4^ cells, were seeded in 48‐well plates and infected with virus at 25 PFU/well for 1.5 h at 37°C, 5% CO_2._ The inoculum was replaced with 1:2 dilutions of the compound to be tested in 1% Carboxymethyl cellulose in DMEM, 2% FBS. Cells were incubated for 3 days then fixed and stained as described above. For IAV, viral yield was evaluated by Tissue Culture Infectious Dose 50 (TCID)_50_/mL.

**Figure 2:**
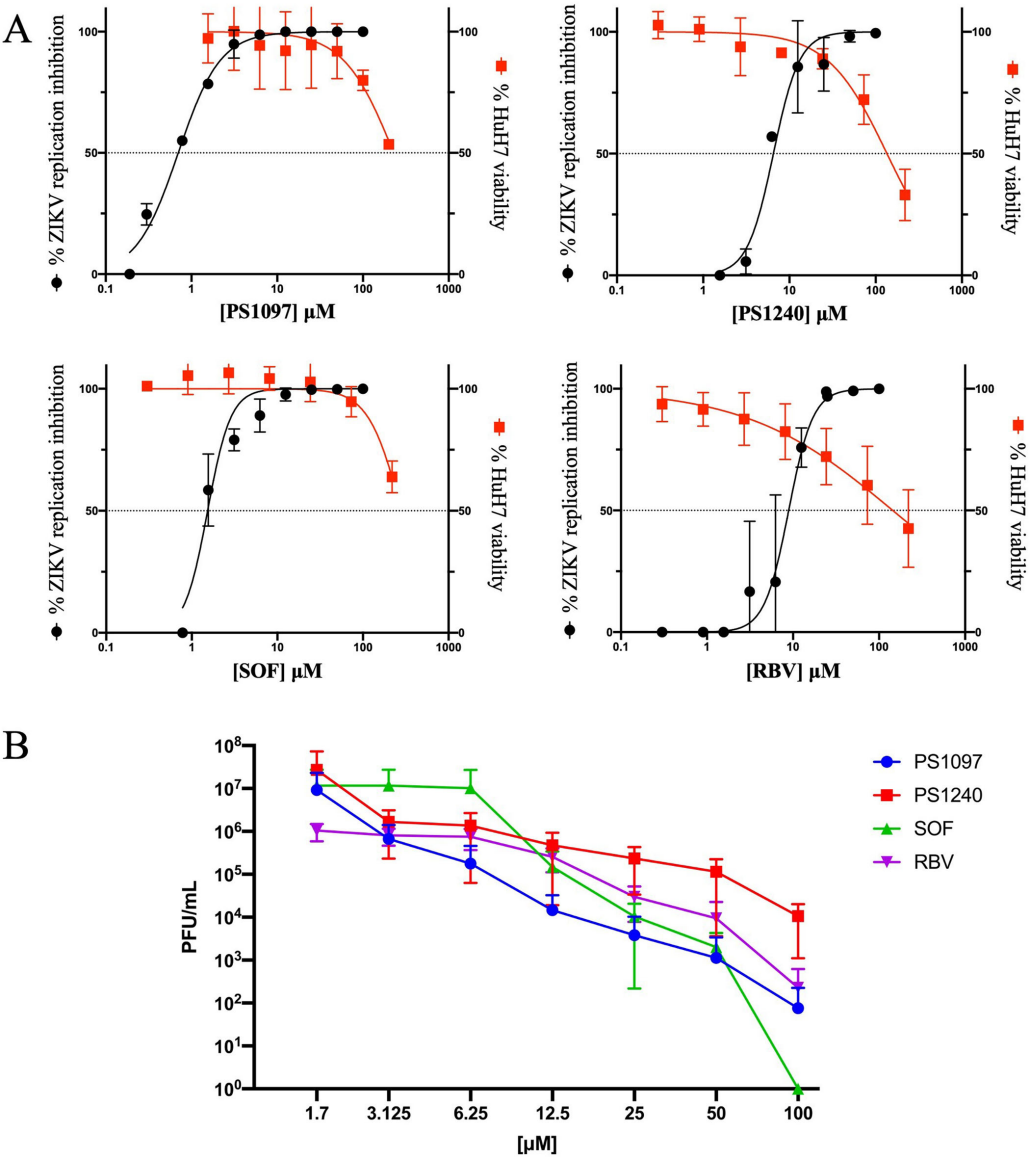
(A) Dose–response curves of the % ZIKV^Br^ replication inhibition (black line) and % cell viability (red line) of the compounds tested and of control drugs SOF and RBV. The antiviral activity of each compound was evaluated on Huh‐7 cells infected with ZIKV^Br^ in the presence of 1:2 dilutions of compounds, starting from 100 μM. Viral yields were determined by titrating supernatants on Vero E6 cells. (B) The same results reported as PFU/mL. Data represent mean values ± SD from at least three independent experiments. RBV, ribavirin; SD, standard deviation; SOF, Sofosbuvir.

EC_50_ values for each compound were calculated by determining viral titers (pfu/ml or TCID_50_/ml) in supernatants of cells infected in the presence of compounds at different concentrations and compared with the DMSO‐treated control; the % inhibition versus log drug concentration was plotted on a graph (Figure 2 and Supporting Information: S1) and EC_50_ values were obtained by nonlinear regression analysis [28]. For clarity, Figure 2B shows the drop in ZIKV titer induced by **PS1097** and **PS1240**.

### 2.5 Determination of Compound Cytotoxicity and Cytotoxic Concentration 50 (CC_50_)

Cytotoxic/cytostatic effects were evaluated in uninfected cells by WST‐8 (Orangu, Cell Guidance Systems): 1 × 10^4^ cells/well in a 96‐well plate were incubated in the presence of threefold serial dilutions of compounds, from 0.30 to 218 μM, for 48 h. Medium was replaced with 50 μL of 10% WST‐8, in DMEM, 2% FBS. Following a 1‐h incubation at 37°C, optical density (OD) in wells was measured at 450 nm using Varioskan LUX (ThermoFisher Scientific). CC_50_ was calculated using the formula:

$$\%\;\text{viability}=100\times ({\text{OD}}_{\text{compound}}/{\text{OD}}_{\text{untreated control}})$$

where OD_compound_ and OD_untreated_ are the OD of the cells treated with compound or DMSO, respectively.

SI was obtained by dividing CC_50_ by EC_50_ for each drug/virus/cell line. Compounds with SI values ≥ 10 were considered to be active in vitro.

The cytostatic effect was assessed by crystal violet [29].

### 2.6 Quantitative Reverse Transcriptase Polymerase Chain Reaction (qRT‐PCR)

Viral RNA was extracted from infected cells with Trizol (Qiagen). qRT‐PCR was performed on ZIKV^Br^ NS1 gene following the instructions of One Step PrimeScript™ III RT‐PCR Kit (Takara Bio), with 0.5 μM primers: **NS1**‐FW 5′‐TGAGATCAACCACTGCAAGY‐3′, **NS1**‐REV 5′‐GCCTTATCTCCATTCCATACCA‐3′; and 0.1 μM probe 5‐FAM/ATCGAGGAATGGTGCTGCAGGGA/3‐BHQ. The mixtures were run in a CFX Connect Real‐Time PCR (Bio‐Rad Laboratories, Hercules, CA, USA) using previously standardized conditions (52°C for 5 min, 95°C for 10 s, 40 cycles of 5 s at 95°C, and 57°C for 30 s). For absolute quantification, standard curves were generated using 10‐fold dilutions of a homemade plasmid at known concentrations. For RTN3, RTN4, and FAM134B mRNA quantification, based on GAPDH, the following primers were used:

RTN3 FW‐5′‐CTTACCTCATCCTGGCTCTTCTC‐3′

RTN3 REV‐5′‐GACAGAGTAATGTCTACGTCCAG‐3′

RTN4 FW‐5′‐ TCTTSSTGCTGCATCTGAGCCT‐3′

RTN4 REV‐5′‐ GCAGTTTCAAGCAGGACAGATGG‐3′

FAM134B FW‐5′‐GTCTCAGAGGTATCCTGGACTG‐3′

FAM134B REV‐5′‐ TTCCTCACTGGGTCGGTCAAGA‐3′

GAPDH FW‐5′‐GTCTCCTCTGACTTCAACAGCG‐ 3′

GAPDH REV‐5′‐ACCACCCTGTTGCTGTAGCCAA ‐3′

### 2.7 Western Blot Analysis

Immunoblotting was performed as described [30]. Briefly, cells were washed in phosphate‐buffered saline (PBS) and lysed using RIPA lysis buffer added with protease and phosphatase inhibitors (Thermo Fisher Scientific). Lysates were added with loading buffer, subjected to sodium dodecyl sulfate‐polyacrylamide gel electrophoresis (SDS‐PAGE) on 12% PAGE gels, and transferred on nitrocellulose (Millipore). The antibodies used (Supporting Information: Table S1) were diluted in 5% Skim milk in PBS, 0.1% Tween 20.

### 2.8 Time‐of‐Addition (TOA) Experiments

12 × 10^4^ Huh‐7 cells per well in a 12‐well plate were infected with ZIKV^Br^ (MOI:1) for 2 h. **PS1097** was added at 6 μM at 0, 2, 6, 12, 24, and 36 h p.i [31]. SOF was used as a reference compound at 15 μM. At 48 h p.i., viral proteins were determined by western blot or by qRT‐PCR in cell lysates.

To monitor intracellular viral RNA production kinetics in untreated cells, these were infected as described above for 2 h. After removing the inoculum, assay medium was added, then cells were collected at the time points of the TOA assay. Viral RNA replication was monitored as described above in cell lysates.

### 2.9 Selection of Viral Mutants

Huh‐7 cells, 6 × 10^4^ per well in a 24‐well plate, were infected with ZIKV^Br^ (MOI:1) for 2 h at 37°C. Virus was then removed and cells were further incubated with 0.6 μM and 1.2 μM **PS1097** for 2 days at 37°C. The supernatants were then titrated by plaque assay and used to infect freshly seeded cells at MOI:1. The remaining supernatant was stored at −80°C. During weekly passaging of the virus, the starting concentration of the compound was gradually increased. In parallel, wild‐type ZIKV^Br^ was passaged using Huh‐7 cells in a similar way to compound‐treated virus.

### 2.10 Synergism Between PS1097 and SOF

Cells were infected with ZIKV^Br^, MOI:1, as described above and treated with serial twofold dilutions of the **PS1097**/SOF combinations (**PS1097** 2.0–0.125 μM; SOF (5.0–0.39 μM, Figure 4). At 48 h p.i., antiviral activities of each combination were evaluated in supernatants as described [28]. Percent inhibition of viral replication was plotted, and drug interaction analyzed by SynergyFinder (https://synergyfinder.fimm.fi) or MatLab (mathworks. com). Synergy scores less than −10 indicated an antagonistic interaction, scores between −10 and 10 an additive effect, and scores greater than 10 a synergistic effect between drugs.

### 2.11 Graphics and Statistics

Graphs and statistical analyses were performed by GraphPad Prism 7 software. Data are shown as mean ± SD or 95% confidence intervals.

## 3 Results

### 3.1 Antiviral Activity Against ZIKV by a Selection of Compounds

This study began by evaluating 24 compounds with known antiviral activity against various viral agents (Supporting Information: Table S2). The compounds active against BVDV were tested against ZIKV because the viruses belong to the same family. Specifically, ZIKV^Br^, considered one of the most pathogenic strains, was used [3]. Two pyrido[2,3‐g] quinoxalinone derivatives, PS1097 and PS1240, emerged as the most active against ZIKV (Figure 1, Table 1 and Supporting Information: S2). SOF, a potent HCV inhibitor exerting antiviral activity against other members of the *Flaviviridae* family, was used as a control [23]. Huh‐7 cells were used, and antiviral activity was tested at a concentration range of 100–1.5 μM by viral yield reduction assays. In parallel, compound toxicity was evaluated on the same cells and at the same compound concentrations. Figure 2A shows the graphs obtained by plotting antiviral activity and cytotoxicity values for the two most active compounds. The data show that the thiophene derivatives **PS1097**: 5‐chloro‐3‐(thiophen‐2‐yl) pyrido[2,3‐*g*] quinoxalin‐2(1*H*)‐one and, to a lesser extent, its analog substituted on N‐1 with a methyl group (**PS1240**) had marked inhibitory activity against ZIKV^Br^ (EC_50_ 0.6 and 2.06 µM, respectively). RBV did not seem to be active against ZIKV^Br^ at nontoxic concentrations (Figure 2), while SOF exhibited an EC_50_ of 1.5 µM and was used as a reference drug thereafter.

Thus, **PS1097** showed the highest antiviral activity against ZIKV^Br^ and was prioritized for further experiments. Being structurally related to **PS1240**, we assumed that the activity of the two compounds would be relying on the same mechanisms (Table 1).

**Table 1:** Antiviral activity of the compounds tested against ZIKV^Br^ on Huh‐7 cells.

Compound	EC_50_ (μM)	CC_50_ (μM)	SI
PS462	> 100	294 (186–464.8)^a^	< 2.9
PS1240	2.06 (1.9–2.2)^a^	293.1 (238.1–360.7)^a^	142.3
PS1097	0.69 (0.6–0.8)^a^	228.9 (150.2–348.7)^a^	331.7
RBV	8.8 (7.2–11)^a^	144 (78–265.9)^a^	16.3
SOF	1.5 (1.4–1.7)^a^	281.7 (221.1–358.8)^a^	184

Abbreviations: CC_50_, cytotoxic concentration 50%; EC_50_, effective concentration 50%; RBV, ribavirin; SI, Selectivity Index; SOF, Sofosbuvir.

^a^ 95% Confidence interval.

### 3.2 Antiviral Activity of PS1097 on Different Viruses and Cell Lines

**PS1097** was tested on other permissive cell lines, including simian Vero E6, Vero TMPRSS, human HMC3, A549, and *Aedes albopictus* C6/36. Viral yield assays were performed if not specified otherwise. EC_50_ values were comparable on human and monkey cells, while slightly lower SI were obtained on Vero E6 cells (due to lower CC_50_). **PS1097** also inhibited ZIKV in insect C6/36 cells, although with an SI roughly 10 times lower, suggesting a conserved mechanism of action across mammalian and insect cells (Table 2).

**Table 2:** Antiviral activity of PS1097 against ZIKV^Br^ in various cell lines and against other viral species and strains.

	EC_50_ (μM)	CC_50_ (μM)	SI^a^
Cell line			
Huh‐7	0.69 (0.6–0.8)^b^	228.9 (150.2–348.7)^b^	331.7
VeroE6	1.34 (0.9–1.8)^b^	126.5 (63.6–252.5)^b^	94.4
Vero TMPRSS	2.66 (1.2–6.1)^b^	86.15 (72.6–102.2)^b^	32.4
HMC3	>100	294 (186–464.8)^b^	< 2.9
C6/36	5.51 (4.5–6.8)^b^	150.1 (124.9–180.4)^b^	27.2
A549	44.56 (32.1–64.5)^b^	130 (100.2–184.8)^b^	2.9
Virus strain (cell line)			
ZIKV^Ug^ (Huh‐7)	1.29 (1.1–1.5)^b^	228.9 (150.2–348.7)^b^	177.4
USUV (Huh‐7)	0.86 (0.5–1.4)^b^	228.9 (150.2–348.7)^b^	266.2
WNV (Huh‐7)	1.15 (1–1.5)^b^	228.9 (150.2–348.7)^b^	199
SARS‐CoV‐2^Mi^ (Vero TMPRSS)	0.1 (0.02–0.4)^b^	436.1 (113.7–1674)^b^	4361
CVB5 (A549)	4.47 (2.8–7.6)^b^	218.2 (200–241.4)^b^	48.8
CHIKV (Huh‐7)	4.4 (3.3–5.9)^b^	228.9 (150.2–348.7)^b^	52
VSV (Huh‐7)	> 50	228.9 (150.2–348.7)^b^	na
VV‐IND‐G (Huh‐7)	1.73 (0.6–4.3)^b^	228.9 (150.2–348.7)^b^	132.3
HSV2 (A549)	> 50	218.2 (200–241.4)^b^	na
IAV (MDCK)	> 50	nd	na
TOSV (VeroE6)	> 50	574.6 (195.8–1678)^b^	na

Abbreviations: CC_50_, cytotoxic concentration 50%; CHIKV, chikungunya virus; CVB5, Coxsackie B virus, serotype 5; EC_50_, effective concentration 50%; HSV2, herpes simplex virus 2; IAV, influenza A virus; na, no antiviral activity; nd, not determined; TOSV, Toscana virus; USUV, Usutu virus; VV‐IND‐G, vaccinia virus recombinant for VSV‐Indiana G protein; VSV, vesicular stomatitis virus; WNV, West Nile virus; ZIKV, Zika virus.

^a^ Selectivity index (SI) was calculated by dividing CC_50_ by EC_50._

^b^ 95% confidence interval.

To investigate the activity range of **PS1097**, it was tested against another ZIKV strain and other viral species to determine whether it had broad antiviral effects. **PS1097** showed an EC_50_ of 1.29 against the African ZIKV Uganda strain (ZIKV^Ug^), comparable to the one against ZIKV^Br^. It was also effective against other *Flaviviridae* members, including USUV and WNV, with an EC_50_ comparable to ZIKV (Table 2). It also showed an inhibitory effect against SARS‐CoV‐2^Mi^, an early COVID‐19 pandemic variant, though with a higher EC_50_ than against *Flaviviridae* (Supporting Information: Figure S1). Antiviral activity was further tested on viruses from different families by plaque reduction assays. It exhibited activity against CVB5, CHIKV, and VV‐IND‐G (a DNA virus), but no significant activity was observed against ssRNA^‐^ viruses like VSV, TOSV, or Herpes simplex 2 (HSV2). IAV, evaluated by TCID_50_ reduction, was also insensitive (Table 2).

### 3.3 Determination of Viral Protein Synthesis During Treatment With PS1097

**Figure 3:**
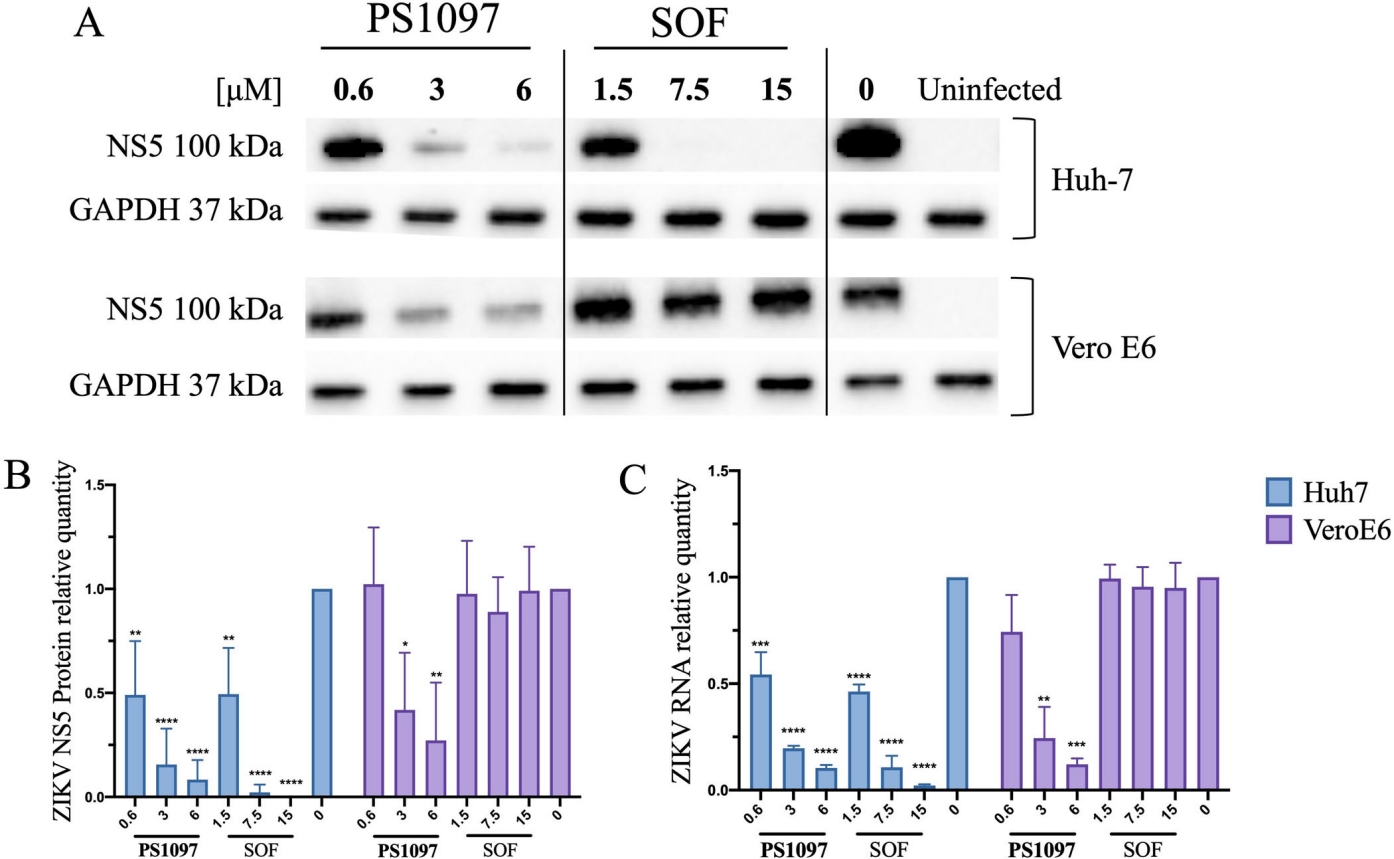
(A) Huh‐7 cells (2 upper panels) or Vero E6 (2 lower panels) were infected with ZIKV, MOI:1, and treated with PS1097 or SOF at the concentrations shown (IC_50_ ×1, ×5, and ×10) for 48 h. Impact of treatment on ZIKV protein synthesis was evaluated on cell extracts analyzed by western blot using anti‐ ZIKV NS5, or anti‐GAPDH as a housekeeping control. (B) Quantitative analysis of the results in A. (C) Viral yield determination by qRT‐PCR quantification of ZIKV genome quantity on supernatants taken from the cells used in western blot relative to untreated infected cells. Statistical analysis was performed using One‐way ANOVA (*p* < 0.01; *α* = 0.05). Data are expressed as mean ± SD, *N* = 4. Antibodies used are shown in Supporting Information: Table S2. ANOVA, analysis of variance; qRT‐PCR, quantitative real‐time polymerase chain reaction; SD, standard deviation; SOF, Sofosbuvir.

To determine whether the reduction in ZIKV yield by **PS1097** was due to inhibiting viral protein synthesis or viral release, Huh‐7 and Vero E6 cells were infected with ZIKV^Br^ and treated with **PS1097** or SOF at 1×, 5×, and 10× their EC_50_ (0.6, 3, 6 μM and 1.5, 7.5, 15 μM, respectively). Viral protein levels in cell lysates were analyzed by immunoblotting (Figure 3A,B), while virion release was assessed by quantifying ZIKV RNA in supernatants (Figure 3C).

**PS1097** downregulated viral protein expression in Huh‐7 cells but did not fully suppress it, whereas SOF completely abolished protein synthesis at 7.5 μM (5× EC_50_) (Figure 3A,B). Interestingly, in Vero E6 cells, SOF had no effect on viral protein content, as previously reported [32], while **PS1097** consistently reduced protein levels, as in Huh‐7 cells. Viral RNA levels in supernatants closely mirrored intracellular viral protein quantification (Figure 3B,C).

These findings indicate that, in both cell lines, PS1097 reduced ZIKV yield primarily by impairing viral protein synthesis rather than by blocking virion release.

### 3.4 TOA and Synergism Experiments

**Figure 4:**
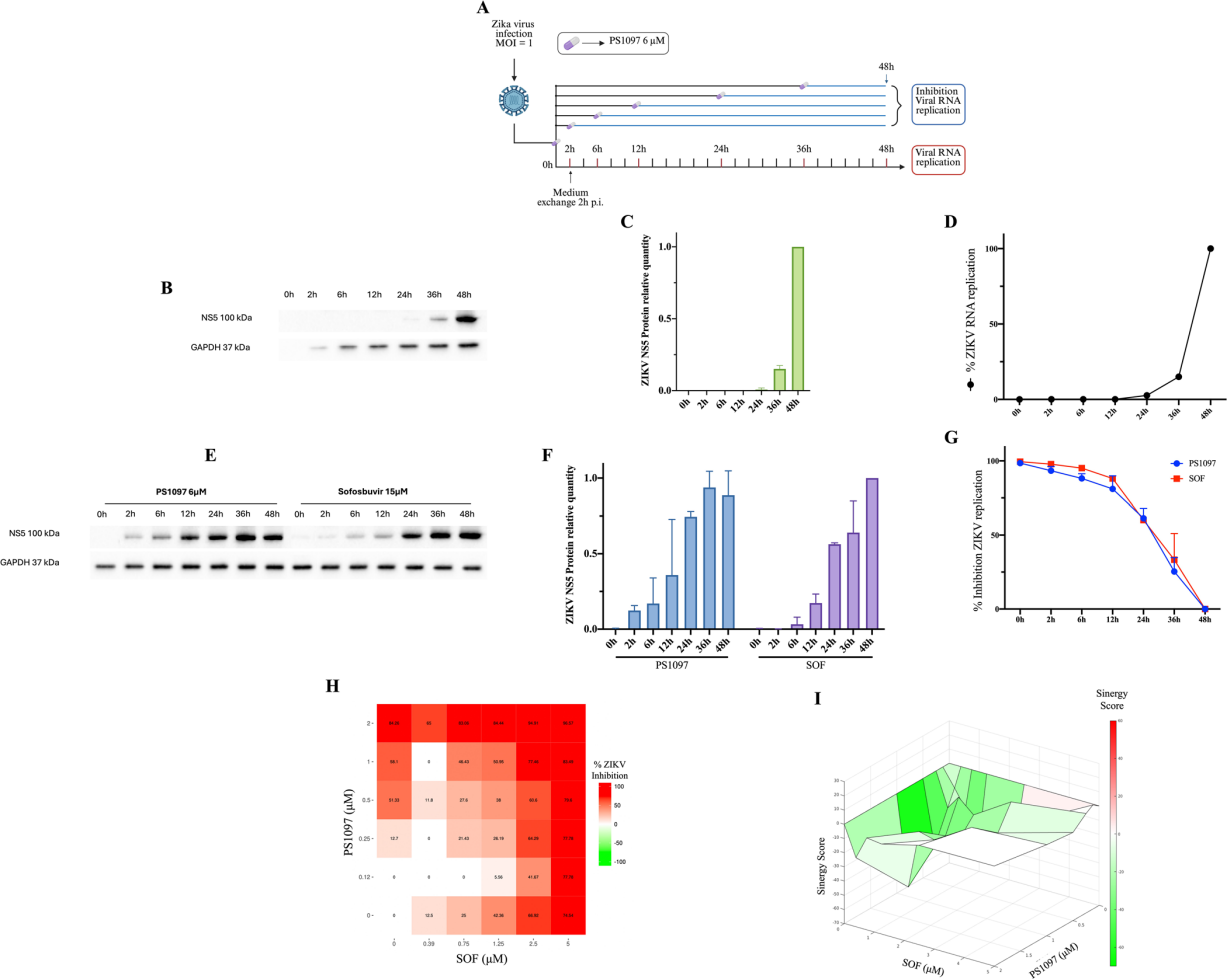
(A) Experimental design of the Time Of Addition (TOA) assay for PS1097 and SOF. (B) ZIKV replication kinetics was determined on Huh‐7 cells infected at MOI 1for 2 h by western blot cell lysates prepared at the times shown. ZIKV NS5 and GAPDH were monitored with the antibodies in Supporting Information: Table S1. (C) Quantitative analysis of the results in B). (D) qRT‐PCR quantification of ZIKV RNA was carried out in ZIKV‐infected cells lysed at the times indicated on the x axis. (E) western blot of lysates of Huh‐7 cells harvested at 48 h p.i. where treatment with PS1097 or SOF was started as by A). (F) Quantitative analysis of the results in E). (G) ZIKV RNA was quantified in lysates of cells harvested at 48 h p.i. where treatment with **PS1097** (red line) or SOF (green line) was started at the times p.i. indicated on the x axis. (H) Combined antiviral activity of **PS1097** with SOF against ZIKV. Huh‐7 cells were infected with ZIKV then treated with **PS1097** or SOF, alone or in combination, at the indicated concentrations. After 48 h p.i., supernatants were harvested and titrated for ZIKV yield. Two‐dimensional representation of dose–response interaction matrix was determined in synergyfinder.org. Color gradient indicates viral yield inhibition score (red‐highest score). (I) Three‐dimensional surface plot representing synergy score (Z axis) for each compound combination. X axis: SOF up to 5 μM, Y axis: **PS1097** up to 2 μM. Green: antagonistic effect, red: synergistic effect. Statistical analysis was performed using One‐way ANOVA (*p* < 0.01; α = 0.05). Data are expressed as mean ± SD, *N* = 3. ANOVA, analysis of variance; qRT‐PCR, quantitative real‐time polymerase chain reaction; SD, standard deviation; SOF, Sofosbuvir.

To elucidate the mechanism of **PS1097** antiviral action, TOA experiments were conducted (Figure 4A). ZIKV^Br^ replication kinetics was assessed by quantifying viral genomic RNA and protein inside Huh‐7 cells at the times p.i. shown in Figure 4A. Viral RNA was detectable as of ~12 h p.i. (Figure 4D), whereas viral protein was visible after 24 h p.i. (Figure 4B,C). In parallel, cells were exposed to viral inoculum for 2 h. For TOA, **PS1097** or SOF were added at 10× EC_50_ together with the viral inoculum (0 h p.i.) or immediately after virus inoculum removal (2 h p.i.) or at the times p.i. shown in Figure 4A. Then, they were harvested at 48 h p.i. Viral RNA in cells was measured by qRT‐PCR and inhibition relative to DMSO‐treated cells was calculated, as described by [33] (Figure 4G). Viral protein quantification revealed **PS1097** suppressed viral protein expression when added up to 12 h p.i. in a time‐flaviviral and coronaviral dependent way (Figure 4E,F): both **PS1097** and SOF demonstrated the strongest inhibition when added at 0–6 h p.i., indicating that **PS1097** is likely to target the early phase of ZIKV replication, with similar kinetics as SOF.

To explore potential interactions between **PS1097** and SOF, combined treatments were tested. No synergistic effects were observed. At the highest concentrations, a slight antagonistic effect was noted (Figure 4H,I), suggesting **PS1097** may directly or indirectly interfere with SOF.

In summary, **PS1097** is most effective when added until 12 h p.i., sharing a similar timing of action with SOF. However, their combined use suggests potential interference and a different mechanism of action.

### 3.5 PS1097‐Resistant Variants

**Figure 5:**
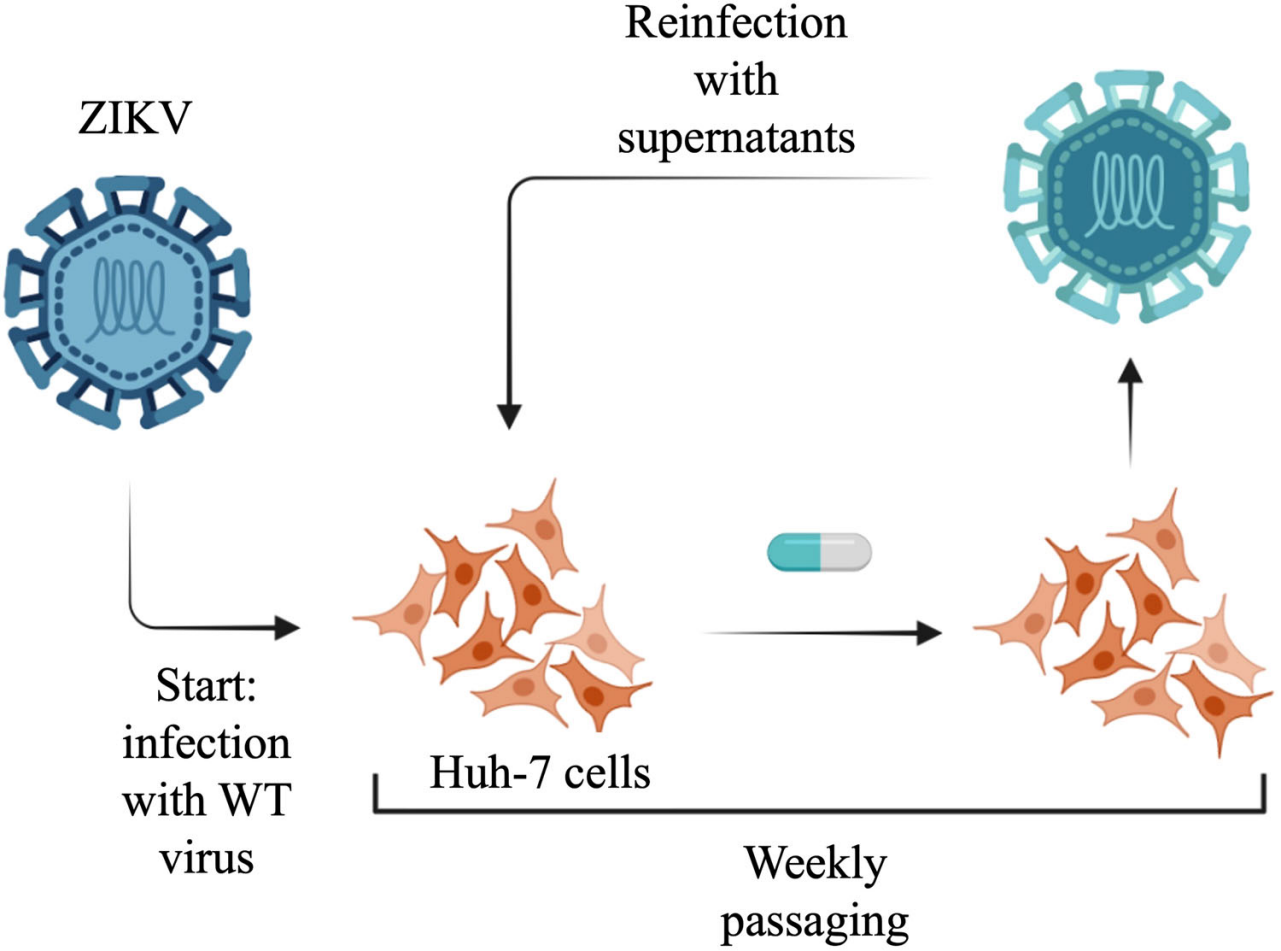
Experimental design for selection of compound‐resistant viral mutants. Huh‐7 cells were infected with ZIKV^Br^ (MOI:1) for 2 h at 37°C, then added with 0.6 μM or 1.2 μM **PS1097** for 48 h at 37°C. The supernatants were then titrated and used to infect freshly seeded cells at MOI:1. During passaging of the virus, the starting concentration of the compound was increased very week.

To identify a viral protein possibly acting as a target for **PS1097** antiviral activity, the possibility to select for **PS1097**‐resistant variants was explored. To this aim, ZIKV^Br^ was passaged in the presence of an initial concentration of 0.6 μM, as graphically described in Figure 5, with the intention of gradually increasing it [33].

At least four attempts were made to isolate mutants starting from wild type virus every time; however, no resistant strains of ZIKV ever adapted to the presence of **PS1097** and viral yield slowly decreased till none was found at the 9th passage (9 weeks), when passaging had to stop.

This suggests that whatever the mechanism targeted by **PS1097**, it is indispensable for viral replication and when it is blocked by the presence of the compound, the virus does not easily mutate to bypass this block. The compound, therefore, exhibits a high barrier to resistance.

### 3.6 PS1097 Causes Potent Downregulation of RTN3 Protein

**Figure 6:**
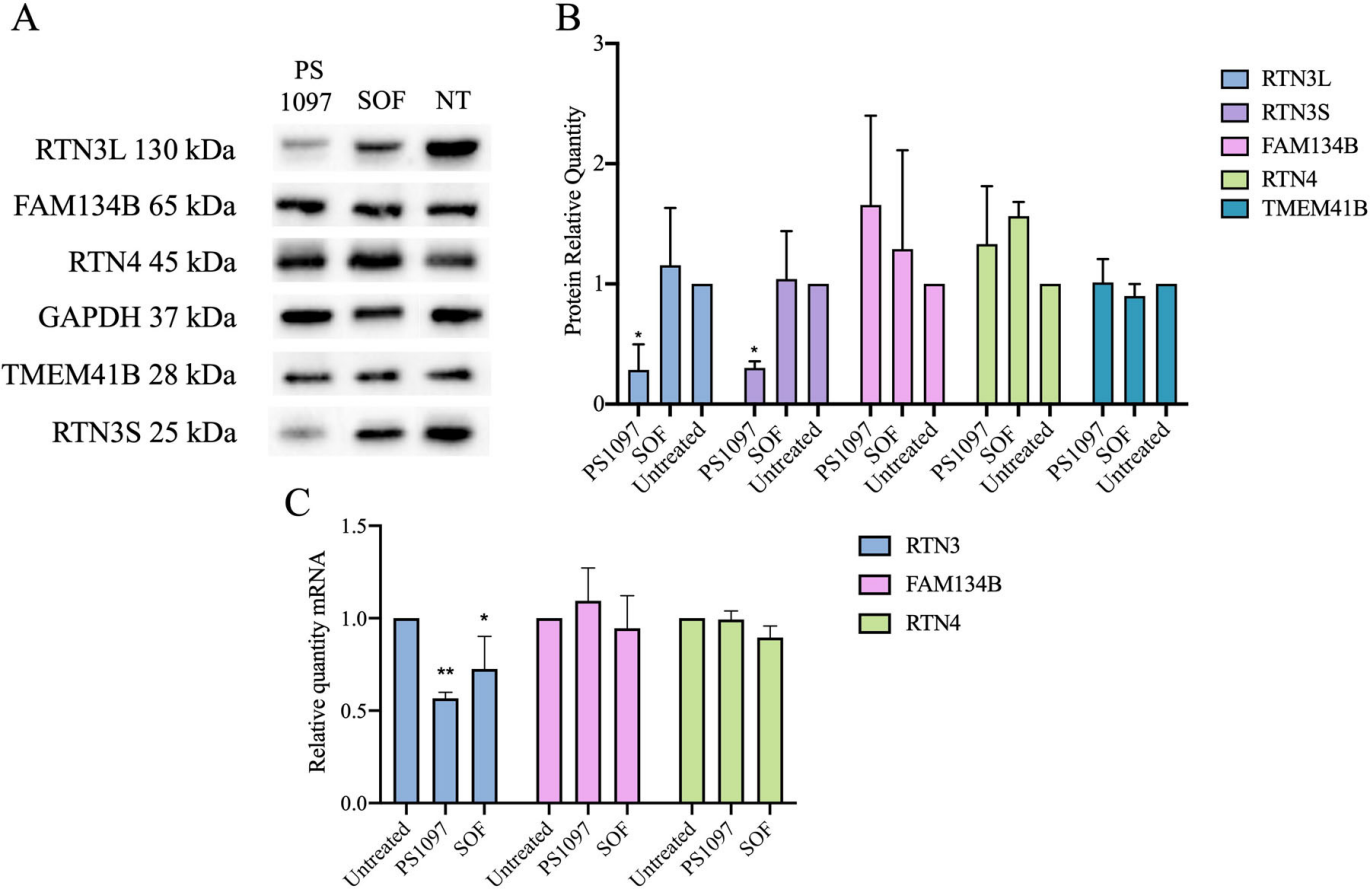
(A) Western blot of Huh‐7 lysed 48 h after treatment with 6 μM PS1097 or 15 μM SOF developed with antibodies against the ER proteins shown on the left or GAPDH as a control. (B) Quantitative analysis of the results in A). (C) qRT‐PCR of RTN3 or RTN4 or FAM134B mRNA in extracts of cells treated with 6 μM **PS1097** or 15 μM SOF for 48 h. Statistical analysis was performed using one‐way ANOVA (*p* < 0.01; *α* = 0.05). Data are expressed as mean ± SD, *N* = 3. Antibodies used are shown in Supporting Information: Table S2. ANOVA, analysis of variance; qRT‐PCR, quantitative real‐time polymerase chain reaction; SD, standard deviation; SOF, Sofosbuvir.

Given the broad range of antiviral activity of **PS1097** (Table 2), it was considered unlikely that the compound could block proteins from totally different viral species. Instead, an indirect activity of **PS1097** acting on a cellular protein important in the replication of a variety of viruses was hypothesized. A common feature of the viruses sensitive to **PS1097** is that most of them cause ER rearrangement during the first phases of their replication, with the notable exception of CHIKV, that rearranges its ROs at the plasma membrane [34]. Therefore, attempts were made to visualize whether other ER resident proteins were affected by treatment of cells with **PS1097**. The proteins selected were: RTN3, placed on the tips of ER tubules [8, 11], RTN4 (*aka* NOGO) playing a pivotal role in ER tubule branching [35] and FAM134B, one of the most important reticulophagy receptors [36]. In addition, TMEM41B was also tested because it was reported to be an important cellular cofactor in flaviviral and coronaviral replication [37]. Huh‐7 cells were treated with 6 μM **PS1097** or, as a negative control, SOF, 15 μM, for 48 h, then they were lysed for western blot. The results, shown in Figure 6, demonstrate that treatment with **PS1097** caused a significant decrease in RTN3 content, whereas FAM134B, RTN4, and TMEM41B were virtually unchanged. To exclude that the effect was due to the antibody used or to differential splicing of the RTN3 mRNA that might cause the elimination of an epitope recognized by the other antibody, two different antibodies were used, one detecting the N‐terminus (RTN3L) and the other the C‐terminus of RTN3 (RTN3S) and similar results were obtained (Figure 6A,B).

To evaluate whether reduced RTN3 mRNA transcription contributes to the observed decrease in RTN3 protein levels, qRT‐PCR was performed on cell lysates to quantify mRNA levels for RTN3, RTN4, and FAM134B following treatment with PS1097 or SOF. As shown in Figure 6C, PS1097 treatment led to an approximately 50% reduction in RTN3 mRNA, while SOF treatment resulted in a roughly 25% decrease. However, since the modest reduction in RTN3 mRNA following SOF treatment did not affect RTN3 protein levels, it is unlikely that the 50% decrease in RTN3 mRNA alone accounts for the marked reduction in protein levels observed with PS1097 treatment. Nonetheless, this decrease may contribute to the antiviral activity of PS1097, as RTN3 has been shown to be essential for ZIKV replication [15].

## 4 Discussion

Quinoxaline‐based compound **PS1097** was found to inhibit BVDV replication [18, 19]. In this study, its antiviral activity was investigated against ZIKV, a human virus from the *Flaviviridae* family, to which BVDV also belongs. For comparison, SOF and RBV were included because these drugs are used in the clinics against HCV, another *Flaviviridae* family member from the *Hepacivirus* genus. SOF has shown remarkable activity against ZIKV and was selected as a control [23, 38]. **PS1097** exhibited anti‐ZIKV activity with an EC_50_ of 0.6 μM, in the range of SOF (1.5 μM) and much higher that RBV (8.8 μM). To shed light into the mechanism of antiviral activity of **PS1097**, its specificity was tested against multiple viruses and found to be broadly effective, with similar potency against several members of the *Flavivirus* genus, including WNV, USUV, and two ZIKV strains. Interestingly, **PS1097** displayed even greater efficacy against SARS‐CoV‐2 (EC_50_ 0.1 μM) than against Flaviviruses (EC_50_ around 0.7 μM). Additionally, it showed a certain degree of antiviral activity against CHIKV (a ssRNA+ virus, EC_50_ 4.4 μM) and CVB5 (a ssRNA+ virus, EC_50_ 4.47 μM), as well as against VV, a DNA virus that replicates in the cytoplasm (EC_50_ 1.73 μM, Table 2). The broad activity of **PS1097** against diverse viruses suggests it may act indirectly by targeting a cellular factor required for replication by multiple viruses, rather than directly binding viral proteins. A direct action on such a diverse array of viral species, including a DNA virus lacking an RNA‐dependent RNA polymerase, seems unlikely. Its activity was therefore tested on different cell lines; unlike SOF, which does not inhibit ZIKV infection in Vero E6 monkey cells, **PS1097** effectively inhibited ZIKV replication across most cell types tested, although with different EC50. The reason why the antiviral activity of PS1097 differs widely in cell lines is not known at present. HMC3, where PS1097 seems to be less effective, are less susceptible to ZIKV infection. Both HMC3 and A549 are of human origin, deriving from microglia and respiratory tissue, respectively, while Huh7 are of hepatic background, therefore one can expect the latter to be richer in lipidic membranes and with an expanded ER. A combination of poor susceptibility and different histological origin may explain why PS1097 has a less pronounced antiviral activity on HMC3 and A549.

Cell types where PS1097 has exhibited activity include human, monkey, and insect cells. This suggests that **PS1097** targets a mechanism conserved across eukaryotic cells, down to *A. albopictus* cells that were sensitive to **PS1097**. *A. albopictus* genome has been sequenced and it encodes a reticulon‐3 isoform that has a 56% aminoacidic homology to human RTN3 [39].

In TOA experiments, **PS1097** exhibited a similar window of antiviral activity as SOF, suggesting that it acts early in the viral replication cycle, coinciding with the initiation of genome synthesis (Figure 4). **PS1097** may interfere with the reorganization of the ER and/or other membranes—a key process in the formation of ROs used by positive‐sense single‐stranded RNA (ssRNA+) viruses during the early stages of infection. These viruses are known to replicate within ROs, which are specialized structures generated through the remodeling of ER and plasma membranes following the translation of nonstructural proteins [40, 41, 42, 43]. This mechanism could explain why the timing of PS1097‐mediated inhibition closely matches that of SOF, without having any additive or synergistic effect. Consistently with this hypothesis, the lack of antiviral activity of **PS1097** against HSV2 is unsurprising, as HSV2, a nuclear‐ replicating double‐stranded DNA virus, does not require ER membrane reorganization. In contrast, the strong effect that **PS1097** has on VV replication fits our hypothesis: as a *Poxviridae* member, VV replicates in the cytoplasm and relies on both ER and Golgi membrane reorganization to form virosomes. During early replication, ER‐derived crescent membranes wrap nascent virions in the intermediate compartment between the ER and the trans‐Golgi network, a step critical for efficient VV DNA replication [44]. Further reinforcing this interpretation, PS1097 showed no antiviral activity against three ssRNA– viruses —VSV, IAV, and TOSV—all of which do not require membrane reorganization to form ROs [45, 46]. For instance, VSV is known to rely on phase separation to form compartments that concentrate viral components during assembly, bypassing the need for organelle‐like structures [46].

Aktepe et al. [15] recently demonstrated that silencing RTN3L expression impairs the ability of ZIKV and other flaviviruses to remodel ER membranes, leading to attenuated replication. Building on these findings, the impact of **PS1097** on RTN3 expression was investigated as part of exploring its antiviral mechanism. Treatment with **PS1097** resulted in a marked reduction in RTN3 protein levels, while the expression of other ER‐resident proteins—namely RTN4, FAM134B, and TMEM41B—remained largely unaffected. This selective downregulation of RTN3 may partly explain PS1097's antiviral activity against ZIKV and West Nile virus (WNV).

To our knowledge, this is the first small molecule influencing RTN3 expression. Only silencing with siRNA was evaluated and the proposed link between RTN3 downregulation and flavivirus inhibition, suggested by Aktepe et al., supports the hypothesis that membrane reshaping processes could be a target of PS1097 [14]. Notably, RTN3 has also been implicated in the replication of Enterovirus 71 [13] and SARS‐CoV‐2 [12]. Consistent with these roles, PS1097 demonstrated antiviral efficacy against coxsackievirus B5 (CVB5) and SARS‐CoV‐2 in our assays. However, it is important to note that RTN3 may not be the sole target of PS1097, nor the only factor contributing to its broad antiviral activity.

The mechanism underlying the selective downregulation of RTN3 by PS1097 remains unclear. Although a partial reduction in RTN3 mRNA was observed, transcriptional downregulation alone does not appear sufficient to explain the significant depletion of RTN3 protein. One possibility is that **PS1097** induces reticulophagy, a selective autophagic degradation of ER components, but further studies are required to elucidate the pathways responsible for RTN3 suppression.

## Author Contributions

**Erika Plicanti:** conceptualization, investigation, methodology, writing. **Andrea Deiana:** methodology, investigation. **Silvia Nottoli:** investigation, formal analysis. **Giulia Lottini:** formal analysis, methodology. **Roberta Ibba:** conceptualization, data curation, writing – review and editing. **Sandra Piras:** methodology, investigation. **Carlo Di Marzo:** methodology. **Silvia Vegni:** methodology. **Michele Lai:** funding acquisition, supervision. **Mauro Pistello:** writing – review and editing, funding acquisition. **Antonio Carta:** conceptualization, writing – review and editing, funding acquisition. **Giulia Freer:** conceptualization, writing – review and editing, funding acquisition.

## Conflicts of Interest

The authors declare no conflicts of interest.

## Supporting information


**Appendix A. Supporting data:** Supporting data associated with this article can be found in the online version.

**Scheme S1:** Synthetic route used to obtain key intermediate **6**. Reagents and conditions: *i)* Acetic anhydride, RT 2 h; *ii)* H_2_SO_4_/KNO_3_, 0°C 4 h; *iii)* H_2_SO_4_, 100°–110°C 2 h; i*v)* Glycerol 98% H_2_SO_4_, As_2_O_3_ × 3H_2_O, 110°C 2 h; *v)* NH_3_/EtOH, 150°C 48 h; *vi*) Methylhydrazine, EtOH, 150°C 48 h. **Scheme S2:** Synthetic route performed to gain the final compounds PS462, PS**1097** and PS1240. Reagent and conditions**:** *i)* H_2_SO_4_ 10%, 65°C, 6h; *ii)* (CH_3_O)_2_SO_2_, DMF, Cs_2_O_3_, 60°C 16 h. **Figure S1:** Dose‐response curves of the % SARS‐CoV‐2Mi replication inhibition (black line) and % cell viability (red line) of the compounds tested. The antiviral activity of each compound was evaluated on Vero‐TMPRSS cells infected with SARS‐CoV‐2Mi in the presence of 1:2 dilutions of compounds, starting from 100 μM. Viral yields were determined by titrating supernatants on Vero‐TMPRSS cells. **Table S1:** Antibodies used in this study. **Table S2:** Anti‐BVDV and ‐CVB5/CVB2 activity (EC_50_) and cytotoxicity (CC_50_) values of 24 molecules selected from an in‐house library and their newly tested antiviral activity against ZIKV and cytotoxicity in Huh‐7 cells.

## Data Availability

The data that support the findings of this study are available from the corresponding author upon reasonable request.
