# Supplementary material for: A Pyrido‐Quinoxaline Derivative That Downregulates Reticulon 3 Protein Exhibits Potent Antiviral Activity Against Zika Virus
Source: J Med Virol. 2025 Sep 18;97(9):e70605. doi: 10.1002/jmv.70605 (PMC12445440; doi:10.1002/jmv.70605)
Supplement: Supplementary file 1 — Scheme S1: Synthetic route used to obtain key intermediate 6. Reagents and conditions: i) Acetic anhydride, RT 2 h; ii) H2SO4/KNO3, 0°C 4 h; iii) H2SO4, 100°–110°C 2 h; iv) Glycerol 98% H2SO4, As2O3 × 3H2O, 110°C 2 h; v) NH3/EtOH, 150°C 48 h; vi) Methylhydrazine, EtOH, 150°C 48 h. Scheme S2: Synthetic route performed to gain the final compounds PS462, PS1097 and PS1240. Reagent and conditions: i) H2SO4 10%, 65°C, 6h; ii) (CH3O)2SO2, DMF, Cs2O3, 60°C 16 h. Figure S1: Dose‐response curves of the % SARS‐CoV‐2Mi replication inhibition (black line) and % cell viability (red line) of the compounds tested. The antiviral activity of each compound was evaluated on Vero‐TMPRSS cells infected with SARS‐CoV‐2Mi in the presence of 1:2 dilutions of compounds, starting from 100 μM. Viral yields were determined by titrating supernatants on Vero‐TMPRSS cells. Table S1: Antibodies used in this study. Table S2: Anti‐BVDV and ‐CVB5/CVB2 activity (EC50) and cytotoxicity (CC50) values of 24 molecules selected from an in‐house library and their newly tested antiviral activity against ZIKV and cytotoxicity in Huh‐7 cells. [file JMV-97-e70605-s001.docx]

**Title:**

***A Pyrido-quinoxaline derivative that downregulates Reticulon 3 protein exhibits potent antiviral activity against Zika virus***

Erika Plicanti^a, b#^, Andrea Deiana^c#^, Silvia Nottoli^a#^, Giulia Lottini^a#^ Roberta Ibba^c^, Sandra Piras^c^, Carlo Di Marzo^a,c^, Silvia Vegni^a^, Michele Lai^a^, Mauro Pistello^a, d^, Antonio Carta^c#^, Giulia Freer^a#^

**SUPPLEMENTARY MATERIAL**

Content:

- Synthetic routes
- Compound characterization
- Table S1
- Table S2
- Figure S1

**Synthetic routes**

Compounds were synthesized using an updated version of the previously reported method [1–3].


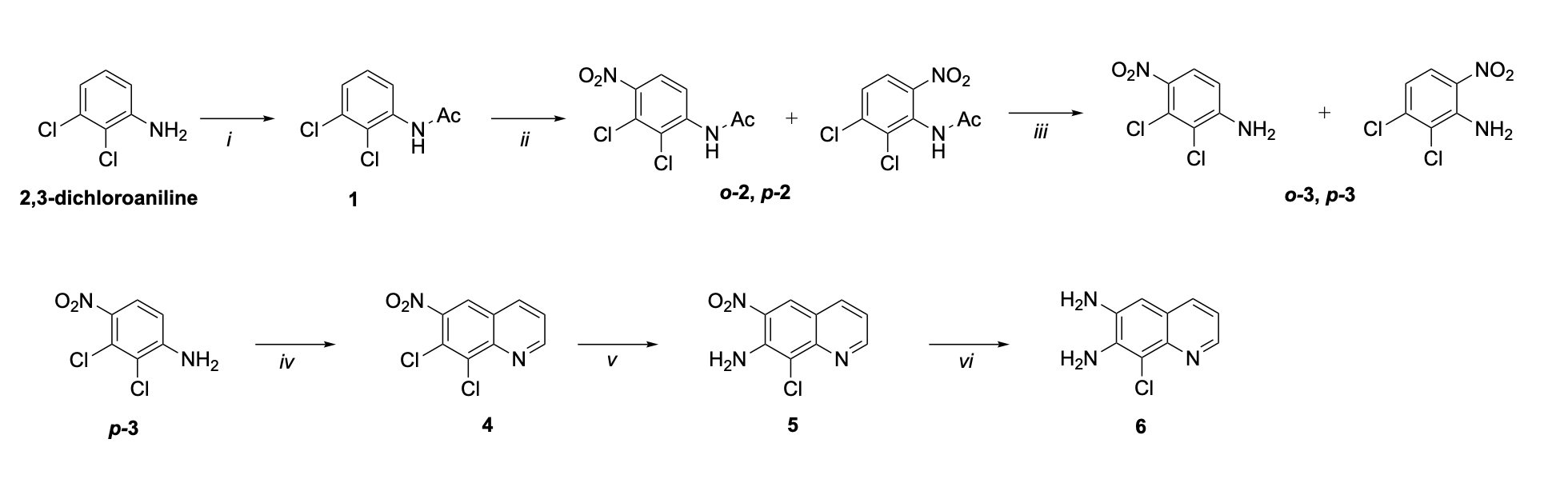


**Scheme S1.** Synthetic route used to obtain key intermediate **6**. Reagents and conditions: *i)* Acetic anhydride, RT 2 h; *ii)* H_2_SO_4_/KNO_3_, 0°C 4 h; *iii)* H_2_SO_4_, 100-110°C 2 h; i*v)* Glycerol 98% H_2_SO_4_, As_2_O_3_ × 3H_2_O, 110°C 2 h; *v)* NH_3_/EtOH, 150°C 48 h; *vi*) Methylhydrazine, EtOH, 150°C 48 h.


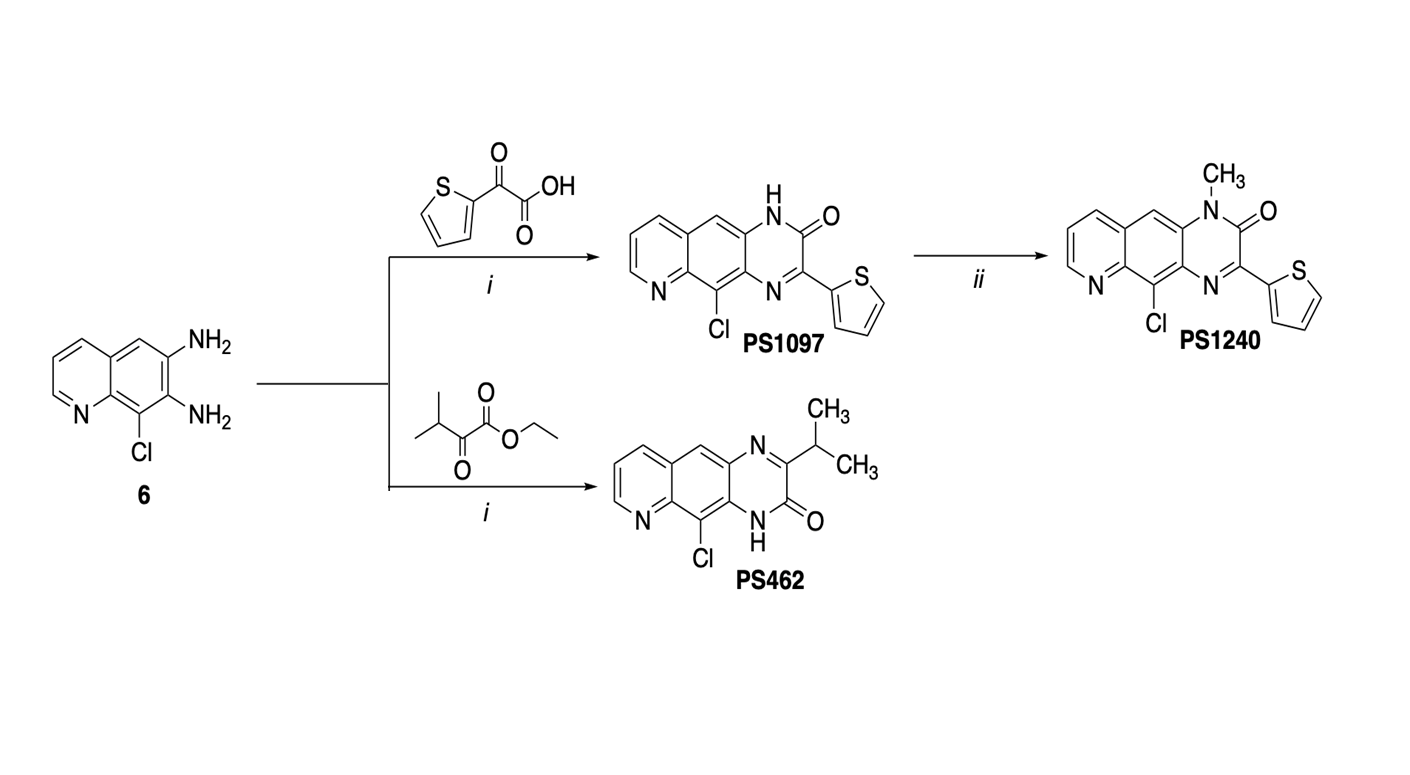


**Scheme S2**. Synthetic route performed to gain the final compounds PS462, PS**1097** and PS1240. Reagent and conditions**:** *i)* H_2_SO_4_ 10%, 65 °C, 6h; *ii)* (CH_3_O)_2_SO_2_, DMF, Cs_2_O_3_, 60°C 16 h.

**Compound characterization**

Resynthesized compounds were purified and characterised, matching the reported data [1,2]. Here yields, TLC Retention factors R*_f_*, and ^1^H-NMR analysis are reported.

**PS462**

Compound was obtained with a yield of 53%; R*_f_* (DCM/MeOH 95/5): 0.54.

^1^H-NMR (400 MHz, DMSO‐*d*_6_) δ: 11.91 (s, 1H, NH), 8.57(dd, *J* = 4.4 and 1.6 Hz,1H, H-7), 8.57 (dd, *J* = 8.4 and 1.6 Hz, 1H, H-9), 8.46 (s, 1H, H-10), 7.61 (dd, *J* = 8.4 and 4.0 Hz, 1H, H-8), 3.54 (m, *J* = 6.8 Hz, 1H, CH), 1.28 (d, *J* = 6.8 Hz, 6H, 2CH_3_).

**PS1097**

Compound was obtained with a yield of 30%; R*_f_* (DCM/MeOH 95/5): 0.45.

^1^H-NMR (400 MHz, DMSO‐*d*_6_) δ: 9.00 (dd, *J* = 4.0 and 1.6 Hz, 1H, H-7), 8.54 (dd, *J* = 4.0 and 1.2 Hz, 1H, H-5′), 8.51 (dd, *J* = 8.4 and 1.2 Hz, 1H, H-9), 7.98 (dd, *J* = 5.2 and 1.2 Hz, 1H, H-3′), 7.71 (s, 1H, H-10), 7.63 (dd, *J* = 4.4 and 8.8 Hz, H-8), 7.32 (dd, *J* = 3.6 and 4.8 Hz, 1H, H-4′).

**PS1240**

Compound was obtained with a yield of 31%; R*_f_* (DCM/MeOH 95/5): 0.87.

^1^H-NMR (400 MHz, DMSO‐*d*_6_) δ: 9.10 (dd, *J* = 4.0 and 1.6 Hz, 1H, H-7), 8.60 (d, *J* = 4.0 Hz, 1H, H-5′), 8.59 (dd, *J* = 4.0 and 8.0 Hz, 1H, H-9), 8.14 (s, 1H, H-10), 8.04 (dd, *J* = 4.8 and 1.2 Hz, 1H, H-3′), 7.75 (dd, *J* = 4.0 and 8.4 Hz, H-8), 7.38 (dd, *J* = 5.2 and 4 Hz, 1H, H-4′), 3.86 (s, 3H, CH_3_).

**Table S1. Antibodies used in this study.**

| **Antigen** | **Host species** | **Manufacturer** | **Cat. #** | **Dilution** |
| --- | --- | --- | --- | --- |
| ZIKV Capsid | rabbit | GeneTex | GTX133317 | 1:1000 |
| ZIKV NS5 | rabbit | GeneTex | GTX 133328 | 1:1000 |
| GAPDH | mouse | Invitrogen | MA5-15738 | 1:1000 |
| RTN3, N-terminus | rabbit | Genetex | GTX131091 | 1:1000 |
| RTN3 C-terminus, | mouse | Santa Cruz | sc-374599 | 1:1000 |
| RTN4 | mouse IgM | Santa Cruz | sc-271878 | 1:1000 |
| FAM134 | rabbit | Proteintech | 21537-1-AP | 1:1000 |
| TMEM41B | rabbit | My Biosource | MBS154820 | 1:1000 |
| rabbit IgG | Goat, HRPO | Merck | A0545 | 1:20.000 |
| mouse IgG | Rabbit, HRPO | Merck | A9044 | 1:20.000 |

**Table S2**. Anti-BVDV and -CVB5/CVB2 activity (EC_50_) and cytotoxicity (CC_50_) values of 24 molecules selected from an in-house library and their newly tested antiviral activity against ZIKV and cytotoxicity in Huh-7 cells.

| **Label – (previously published as)** | **Previously published data** | | | | | **This paper data** | | |
| --- | --- | --- | --- | --- | --- | --- | --- | --- |
|  | **CC_50_ Vero76 (μM)** | **EC_50_ BVDV (μM)** | **EC_50_ CVB5 (μM)** | **EC_50_ CVB2 (μM)** | **Ref.** | **CC_50_ Huh-7 (μM)** | **EC_50_ ZIKV (μM)** | **SI** |
| **PS462 – (4h)** | >100 | 5 | Na | Na | [2] | 294 | 100 | 3 |
| **PS1000 – (2h)** | >100 | 1.2 | Na | Na | [2] | 393.4 | 1.45 | 271 |
| **PS1001 – (2i)** | >100 | 10 | Na | Na | [2] | 126.5 | 1.34 | 94 |
| **PS1067 – (3e)** | >100 | 18 | Na | Na | [2] | 150.1 | 5.51 | 27 |
| **PS1076 – (6b)** | MDBK: >100 | 0.3 | Na | Na | [4] | 86.15 | 2.66 | 32 |
| **PS1086 – (16)** | 30 | 25 | Na | 2 | [3] | Na | Na | Na |
| **PS1097 – (4)** | >100 | 5 | Na | Na | [1] | 228.9 | 0.69 | 331.7 |
| **PS1100 – (18)** | 35 | Na | Na | 2 | [3] | Na | Na | Na |
| **PS1101 – (19)** | 90 | 9 | Na | Na | [3] | Na | Na | Na |
| **PS1240 – (18)** | >100 | 2 | Na | Na | [1] | 293.1 | 2.06 | 142.3 |
| **SMA14 – (5h)** | >100 | Na | Na | 4 | [5] | Na | Na | Na |
| **SMA19 – (5i)** | >100 | Na | Na | 11 | [5] | Na | Na | Na |
| **SMA20 – (5j)** | >100 | Na | Na | 9 | [5] | 178.85 | 2.6 | 69 |
| **SMA87 – (4c)** | >100 | Na | 9 | Na | [6] | 183.4 | 7.88 | 23 |
| **SMA115 – (5g)** | >100 | Na | 8 | Na | [6] | 110.4 | 21.16 | 5 |
| **SMA187 – (100b)** | >100 | Na | 50 | Na | [7] | 72.89 | 10.55 | 7 |
| **SMA189 – (99b)** | >100 | Na | 16 | Na | [7] | Na | Na | Na |
| **SMA292 – (43a)** | >100 | Na | 9 | Na | [7] | 115.6 | 57.17 | 2 |
| **SMA305 – (41a)** | >100 | Na | 18.5 | Na | [7] | 85.54 | 39.52 | 2 |
| **SMA332 – (9a)** | >100 | Na | 23 | Na | [8] | Na | Na | Na |
| **SMA352 – (48a)** | 3.1 | Na | 52 | Na | [7] | 31.51 | 5.29 | 6 |
| **SMA353a – (4c)** | 37 | Na | 37 | Na | [8] | Na | Na | Na |
| **SMA363a – (4d)** | >100 | Na | 10 | Na | [8] | 57.11 | 7 | 8 |
| **SMA364a – (4a)** | 90 | Na | 13 | Na | [8] | Na | Na | Na |
| **EIDD-1931** |  |  |  |  |  | 147.7 | 1.704 | 87 |
| **RBV** |  |  |  |  |  | 144 | 8.8 | 16 |
| **SOF** |  |  |  |  |  | 281.7 | 1.53 | 184 |
|  |  |  |  |  |  |  |  |  |

EC_50_: Effective Concentration 50%; CC_50_: Cytotoxic Concentration; Na: ‘no detected antiviral activity’; EIDD-1931: β-D-N4-hydroxycytidine; RBV: Ribavirin; SOF: Sofosbuvir.

**Figure S1**


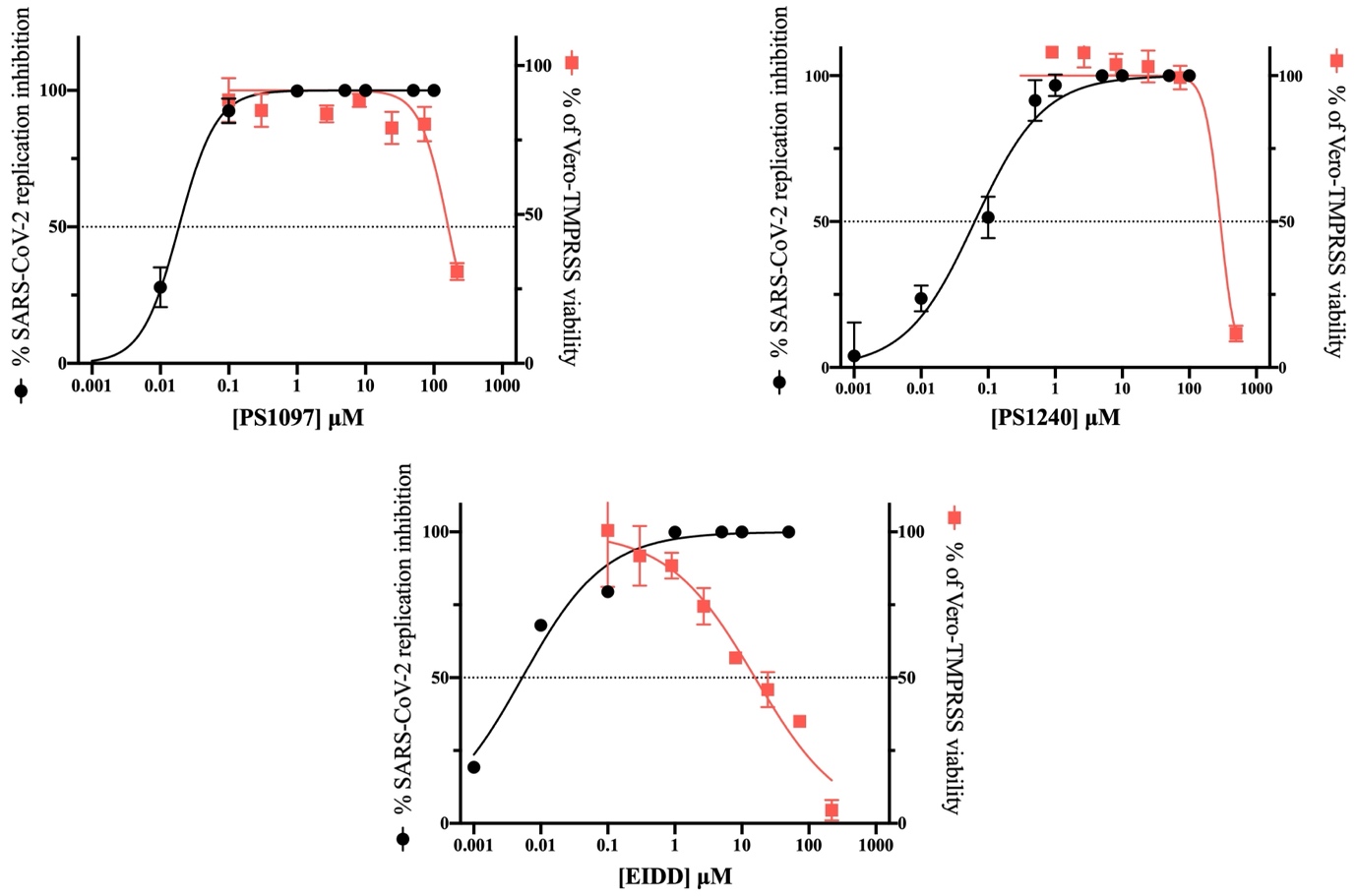


**Fig. S1**. Dose-response curves of the % SARS-CoV-2^Mi^ replication inhibition (black line) and % cell viability (red line) of the compounds tested. The antiviral activity of each compound was evaluated on Vero-TMPRSS cells infected with SARS-CoV-2^Mi^ in the presence of 1:2 dilutions of compounds, starting from 100 μM. Viral yields were determined by titrating supernatants on Vero-TMPRSS cells.

Data represent mean values ± SD from at least three independent experiments.

**References**

[1] Briguglio I, Loddo R, Laurini E, et al. Synthesis, cytotoxicity and antiviral evaluation of new series of imidazo[4,5-g]quinoline and pyrido[2,3-g]quinoxalinone derivatives. Eur J Med Chem 2015;105:63–79. https://doi.org/10.1016/j.ejmech.2015.10.002.

[2] Carta A, Briguglio I, Piras S, et al. Quinoline tricyclic derivatives. Design, synthesis and evaluation of the antiviral activity of three new classes of RNA-dependent RNA polymerase inhibitors. Bioorg Med Chem 2011;19:7070–84. https://doi.org/10.1016/j.bmc.2011.10.009.

[3] Carta A, Loriga M, Paglietti G, et al. Design, synthesis, and preliminary in vitro and in silico antiviral activity of [4,7]phenantrolines and 1-oxo-1,4-dihydro-[4,7]phenantrolines against single-stranded positive-sense RNA genome viruses. Bioorg Med Chem 2007;15:1914–27. https://doi.org/10.1016/j.bmc.2007.01.005.

[4] Carta A, Briguglio I, Piras S, et al. A combined in silico / in vitro approach unveils common molecular requirements for efficient BVDV RdRp binding of linear aromatic N-polycyclic systems. Eur J Med Chem 2016;117:321–34. https://doi.org/10.1016/j.ejmech.2016.03.080.

[5] Carta A, Loriga M, Piras S, et al. Synthesis and Anti-Picornaviridae In Vitro Activity of a New Class of Helicase Inhibitors the N,N-bis[4-(1H(2H)-benzotriazol-1(2)-yl)phenyl] alkyldicarboxamides. Med Chem (Los Angeles) 2007;3:520–32. https://doi.org/10.2174/157340607782360308.

[6] Piras S, Corona P, Ibba R, et al. Preliminary Anti-Coxsackie Activity of Novel 1-[4-(5,6-dimethyl(H)- 1H(2H)-benzotriazol-1(2)-yl)phenyl]-3-alkyl(aryl)ureas. Med Chem (Los Angeles) 2020;16:677–88. https://doi.org/10.2174/1573406416666191226142744.

[7] Ibba R, Corona P, Nonne F, et al. Design, Synthesis, and Antiviral Activities of New Benzotriazole-Based Derivatives. Pharmaceuticals 2023;16:429. https://doi.org/10.3390/ph16030429.

[8] Ibba R, Piras S, Corona P, et al. Synthesis, Antitumor and Antiviral In Vitro Activities of New Benzotriazole-Dicarboxamide Derivatives. Front Chem 2021;9. https://doi.org/10.3389/fchem.2021.660424.
